# Ethanol’s Effect on *Coq7* Expression in the Hippocampus of Mice

**DOI:** 10.3389/fgene.2018.00602

**Published:** 2018-12-04

**Authors:** Diana Zhou, Yinghong Zhao, Michael Hook, Wenyuan Zhao, Athena Starlard-Davenport, Melloni N. Cook, Byron C. Jones, Kristin M. Hamre, Lu Lu

**Affiliations:** ^1^Department of Genetics, Genomics and Informatics, The University of Tennessee Health Science Center, Memphis, TN, United States; ^2^Department of Neurology, Affiliated Hospital of Nantong University, Nantong, China; ^3^Department of Psychology, The University of Memphis, Memphis, TN, United States; ^4^Department of Anatomy and Neurobiology, The University of Tennessee Health Science Center, Memphis, TN, United States

**Keywords:** ethanol, Coenzyme Q, oxidative stress, hippocampus, mouse models, genetics, genomics

## Abstract

Coenzyme Q (CoQ) is a well-studied molecule, present in every cell membrane in the body, best known for its roles as a mitochondrial electron transporter and a potent membrane anti-oxidant. Much of the previous work was done *in vitro* in yeast and more recent work has suggested that CoQ may have additional roles prompting calls for a re-assessment of its role using *in vivo* systems in mammals. Here we investigated the putative role of Coenzyme Q in ethanol-induced effects *in vivo* using BXD RI mice. We examined hippocampal expression of *Coq7* in saline controls and after an acute ethanol treatment, noting enriched biologic processes and pathways following ethanol administration. We also identified 45 ethanol-related phenotypes that were significantly correlated with *Coq7* expression, including six phenotypes related to conditioned taste aversion and ethanol preference. This analysis highlights the need for further investigation of *Coq7* and related genes *in vivo* as well as previously unrecognized roles that it may play in the hippocampus.

## Introduction

**FIGURE 1:**
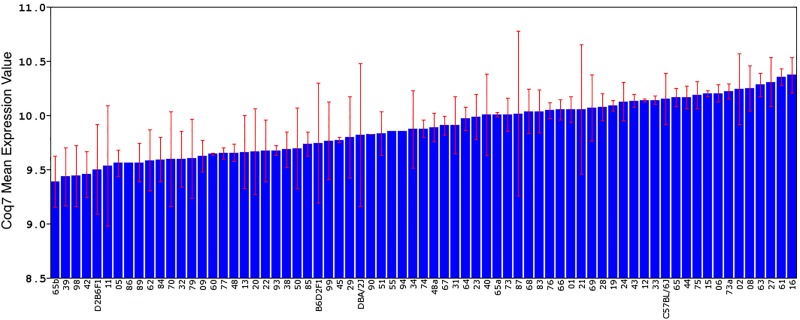
Differential expression of *Coq7* across BXD RI strains rank ordered by expression level. The standard deviation and mean expression of *Coq7* in each strain is shown across the parental DBA/2J and C57BL/6J strains, F1 hybrids, and 67 BXD strains. The *x*-axis represents the mouse strain, while the *y*-axis shows the mean gene expression using the log2 scale.

Coenzyme Q (CoQ or ubiquinol) is a lipophilic molecule present in every cell membrane in the body (Crane, 2001; Turunen et al., 2004). It is best known for its roles as a mitochondrial electron transporter and a potent membrane anti-oxidant (Ernster and Dallner, 1995; Bentinger et al., 2007). CoQ is made up of a benzoquinone ring with an isoprenoid side chain (containing 6–10 units) conserved across species from yeast (as CoQ_6_), to mice (as CoQ_7_), to humans (as CoQ_10_) (Lenaz, 1985). CoQ production in any species is the result of a complex biosynthesis process involving 10 to 15 or more genes (depending on the species) encoding a series of enzymes and non-enzymatic proteins, many of which belong to the *Coq* family of genes (*Coq 1* – *Coq 10A/B*) (Acosta et al., 2016, see their Table 1 and Figure 1 for a complete list). Despite this molecule being characterized and isolated nearly 60 years ago (Festenstein et al., 1955; Wolf et al., 1958; Crane et al., 1989), it continues to remain relevant through ongoing investigations that are fine-tuning its roles in bioenergetics and anti-oxidant defense.

**Table 1:** The single nucleotide polymorphisms (SNPs) in the UTR and coding area of *Coq7* gene.

Chr	Position	Gene	dbSNP	B6	D2	function
7	118509846	Coq7	–	T	C	synonymous_variant
7	118509921	Coq7	rs32487665	T	C	synonymous_variant
7	118510146	Coq7	rs32487669	C	T	stop_gained
7	118525104	Coq7	–	C	T	3_prime_utr_variant
7	118525149	Coq7	rs32485072	T	A	3_prime_utr_variant
7	118525163	Coq7	–	T	C	3_prime_utr_variant
7	118525817	Coq7	rs46657874	C	G	splice_region_variant
7	118525833	Coq7	–	G	A	synonymous_variant
7	118525836	Coq7	–	A	G	synonymous_variant
7	118525851	Coq7	rs13472501	A	G	synonymous_variant
7	118529595	Coq7	rs13459101	G	A	synonymous_variant


Much of the early genetic work regarding CoQ stemmed from submitochondrial fraction studies (Mellors and Tappel, 1966; Landi et al., 1984) and yeast *Saccharomyces cerevisiae* (González-Mariscal et al., 2014), which highlighted CoQ biosynthesis as necessary for mitochondrial antioxidant defense, with less CoQ production resulting in impaired defenses and increased presence of anti-oxidant molecules. But ongoing work has revealed CoQ’s role as an anti-oxidant to be more complex than previously thought. *In vitro* studies using CoQ deficient skin fibroblasts showed severe (<20% of normal) and mild (>60%) CoQ deficiency did not increase reactive oxygen species (ROS) production while moderate deficiency (30–50%) markedly increased ROS production (Quinzii et al., 2008, 2010). Recent *in vivo* studies in CoQ knockout mouse models have been more equivocal, finding that CoQ deficiency does not always directly correspond with ROS production or tissue dysfunction (Quinzii et al., 2013; Licitra and Puccio, 2014; Wang et al., 2015; Luna-Sánchez et al., 2015). This has led some researchers to emphasize the need for better characterization of CoQ’s roles *in vivo* using mammalian models vs. studies *in vitro* or using yeast (Wang and Hekimi, 2016).

In the current study, we use a systems genetics approach to examine the relationship between acute ethanol effects and hippocampal *Coq7* expression in a well characterized genetic population of BXD Recombinant Inbred (RI) mice derived from the C57BL/6J (B6) and DBA/2J (D2) inbred strains, furthering our understanding of CoQ biosynthesis regulation *in vivo*. Currently, there is little evidence connecting *Coq7* and regulation of ethanol responses. However, ethanol metabolism into acetaldehyde is a well understood source of oxidative stress in the brain, causing lipid peroxidation and other oxidative damage to brain tissue (Hipolito et al., 2007; Hernández et al., 2016). This metabolic damage is thought to be warded off by increased gene expression of endogenous anti-oxidants, such as superoxide dismutase (Reddy et al., 1999; Enache et al., 2008). *Coq7* (also known as *mclk-1*) encodes a hydroxylase (coq7p) involved in one of the final steps of CoQ synthesis, conversion of demethoxyubiquinone (DMQ) to CoQ (Acosta et al., 2016). This step in biosynthesis is thought to be the regulated step in CoQ biosynthesis (Marbois and Clarke, 1996; Padilla et al., 2009; Martín-Montalvo et al., 2013; Lohman et al., 2014), making it a prime target to explore how endogenous CoQ production changes in response to acute oxidative stress. Additional evidence suggesting that *Coq7* is important in alcohol responses comes from a study in HXB/BXH RI rats where *Coq7* has been proposed as a candidate gene for alcohol dependency and consumption (Tabakoff et al., 2009).

Previous studies from our lab have shown that gene expression in the hippocampus is particularly sensitive to the effects of acute ethanol (1.8 g/kg) (Urquhart et al., 2016; Baker et al., 2017). Others have also shown that acute ethanol (2.0 g/kg) produces brain region-specific changes in gene expression, including in the hippocampus (Kerns et al., 2005). Here, we demonstrate a positive relationship between acute ethanol ingestion and *Coq7* expression in hippocampus. We also map an expression quantitative trait locus (eQTL) for *Coq7* in BXD RI mice as well as identify pathways associated with *Coq7* and its correlated genes. Through this, we aim to better characterize *Coq7* as a vital gene in the oxidative stress response caused by brain ethanol metabolism.

## Materials and Methods

### BXD Strain and Database Description

The BXD RI mouse strains were derived by crossing the parental strains B6 and D2. The F1 progeny were subsequently intercrossed followed by inbreeding to fix parental genotypes at each locus. The BXD mice are a densely phenotyped and genotyped family and have been used as a genetic reference panel for identifying the genetic basis of phenotypes and diseases, including molecular expression phenotypes, as well as for identifying pathways regulating gene expression. For this study, the dataset of Hippocampus Consortium M430v2 (Jun06) RMA that we generated previously (Overall et al., 2009) was used for genetic mapping, transcript measurement of which was taken from the hippocampus of 67 BXD strains, the parental B6 and D2 strains, and reciprocal F1 hybrids (B6D2F1 and D2B6F1). Detailed information on the strain, sex, age of each animal can be accessed from http://genenetwork.org/webqtl/main.py?FormID=sharinginfo&GN_AccessionId=110. This data set has been uploaded into Gene Expression Omnibus (GEO) with accession number GSE84767 where the data can be downloaded.

### Ethanol Treatment

The BXD parental strains B6 and D2 mice were used for acute ethanol treatment. Ten mice per strain (2∼3 mice per sex) including both males and females at 2–4 months old were divided into two groups: (1) saline group: given an isovolumetric IP injection of saline and (2) ethanol group: treated with an IP injection of 2.0 g/kg i.p., ethanol (12.5% v/v). The dose of 2.0 g/kg of ethanol was chosen to allow for comparisons with other studies in mice seeking to produce genetically based ethanol-sensitive behaviors (Cunningham and Noble, 1992; Cunningham and Prather, 1992; Cunningham, 1995; Risinger and Cunningham, 1995, 1998). Twenty-four hours after treatment, these mice were sacrificed for tissue harvest (Cook et al., 2015).

### Tissue Harvest

The B6 and D2 mice treated with ethanol or saline were anesthetized with an overdose of avertin (1.25% 2,2,2- tribromoethanol and 0.8% tert-pentyl alcohol in water; 0.8–1.0 ml, i.p.) until they were immobile, a period of less than 2 min. After this time mice were sacrificed by cervical dislocation. The hippocampi were harvested according to previously described methods (Lu et al., 2001). The left and right hippocampi were pooled and stored in RNAlater overnight at 4°C, then kept at -80°C until RNA extraction.

### RNA Extraction

RNA was extracted from the hippocampus using RNA STAT-60 (protocols can be found at Tel-Test ^1^) as per the manufacturer’s instructions. A spectrophotometer (Nanodrop Technologies^2^) was used to measure RNA concentration and purity, and the Agilent 2100 Bioanalyzer was used to evaluate RNA integrity. To pass quality control, the RNA integrity values needed to be greater than 8. The majority of samples had values between 8 and 10.

### Gene Expression

The Affymetrix GeneChip^TM^ Mouse Transcriptome Array 1.0 (MTA 1.0) was used to generate gene expression data, from B6 and D2 mice treated with ethanol or saline, according to the manufacturers’ protocol. Affymetrix Expression Console Software was used to identify and remove outlier arrays, and normalize raw data in CEL files using the Robust Multichip Array (RMA) method (Pan et al., 2011). The expression data were then re-normalized using a modified *Z* score described in a previous publication (Bolstad et al., 2003). We calculated the log base 2 of the normalized values, computed *Z* scores for each array, multiplied the *Z* scores by 2, and added an offset of 8 units to each value. This transformation yields a set of *Z*-like scores for each array that have a mean of 8, a variance of 4, and standard deviation of 2. The advantage of this modified *Z* score is that a twofold difference in expression corresponds approximately to a 1 unit change.

### Quantitative RT-PCR

Total RNA from 10 hippocampi per treatment group (both B6 and D2 mice) was used for a quantitative RT-PCR experiment. The gene-specific probe and primer sets for *Coq7* (upstream 5′-tttggaccatagctgcattg-3′, downstream 5′-tgaggcctcttccatactctg-3′) were deduced using Universal Probe Library Assay Design software^3^. *Coq7* mRNA levels were detected and analyzed on a LightCycler 480 System (Roche, Indianapolis, IN, Unites States^3^) under the following cycling conditions: 1 cycle at 95°C for 5 min and then 40 cycles at 95°C for 10 s, 60°C for 30 s, and 72°C for 10 s. The PCR mix contained 0.2 μl of 10 μM primers, 0.1 μl of 10 μM Universal library probe, 5 μl of LC 480 master mix (2×), 2 μl of template cDNA, and RNase-free water to 10 μl. TATA box-binding protein (TBP) was selected as the endogenous quantity control. The relative gene expression of *Coq7* was analyzed with the ΔΔCT method with TBP used as the reference gene for normalization. *Coq7* expression (fold change) in each ethanol treated mouse relative to average of the corresponding control mice was calculated as: Fold Change = 2ˆ-[ΔCT(*Coq7* in each ethanol treated mouse) - (Mean of ΔCT of control mice)].

### Statistical Analysis

The *Coq7* gene expression data from saline or alcohol treatment in B6 and D2 mice were evaluated using the analysis of covariance (ANCOVA) with treatment, strain, and sex as factors, and adjusted for age and body weight.

### Phenotype QTL and Expression QTL (eQTL) Mapping and SNP Analysis

We performed phenotype QTL and eQTL analyses using the WebQTL module on GeneNetwork ^4^ according to our published methods (Chesler et al., 2005). The genome-wide efficient mixed model association algorithm (GEMMA) was used to identify potential eQTLs regulating *Coq7* expression levels and phenotype QTLs near the *Coq7* locus, and to estimate the significance at each location using known genotypic data for those sites. Each of these analyses produced a likelihood ratio statistic (LRS) score, providing us with a quantitative measure of confidence of linkage between the observed phenotypes or expression level of *Coq7* and known genetic markers. The significance of the QTL and eQTLs were calculated using more than 2000 permutations tests. Loci were considered statistically significant if genome-wide *p* < 0.05. Sequence variability between B6 and D2 was then determined using the Sanger mouse SNP database^5^.

### Gene Function Analysis

Prior to gene function analysis, co-expression and literature correlation were performed to filter a list of transcripts correlated with *Coq7* gene expression in the ethanol and saline groups. Co-expression analysis was performed on GeneNetwork. *Coq7* probe set expression was compared to all probe sets on the MTA 1.0 array. Criterion for significant co-expression included average log_2_ probe set expression greater than 7.0, as well as a significant correlation with *Coq7*, indicated by a Pearson product correlation value (*p* < 0.05). We further filtered *Coq7* co-expressed probe sets in the ethanol and saline groups by performing literature correlations using the Semantic Gene Organizer to find the potential biological correlation between *Coq7* and other genes (Homayouni et al., 2004). Genes with higher correlation values (*r* > 0.3) were selected for further analysis. The top 500 genes with significant co-expression (*p* < 0.05) and literature correlations (*r* > 0.45) for the ethanol and saline data sets were then selected and uploaded to Webgestalt^6^ for gene function analyses (Zhang et al., 2005). Enrichment of biological function in the top 500 *Coq7* ethanol and saline co-expression data sets was determined using the hypergeometric test. The *p*-values from the hypergeometric test were automatically adjusted to account for multiple comparisons using the Benjamini and Hochberg correction (Benjamini and Hochberg, 1995). Categories with an adjusted *p*-value of less than 0.05 indicated that the set of submitted genes was significantly over-represented in those categories.

### Phenotype Correlation

We used the BXD phenotype database in our GeneNetwork website^4^ to find alcohol phenotypes highly correlated (Pearson product correlation, *p* < 0.05) with expression of the *Coq7* probe set in hippocampus from naïve BXD mice (Hippocampus Consortium M430v2 (Jun06) RMA data set).

## Results

### *Coq7* Expression Variance Across BXD Mice and Heritability

Only one probe set in the Affymetrix M430 dataset represents the *Coq7* gene (1415556_at), which targets the last five coding exons. *Coq7* expression (log2 scale) varied widely between BXD strains, with a fold-change of 2.57 (Figure 1). BXD65b had the lowest expression (9.38 ± 0.24), and BXD16 had the highest expression (10.70 ± 0.16). There is also a 1.26-fold difference in *Coq7* transcript abundance between B6 (10.15 ± 0.23) and D2 (9.81 ± 0.66).

### eQTL Mapping and Sequence Variants of Coq7

**FIGURE 2:**
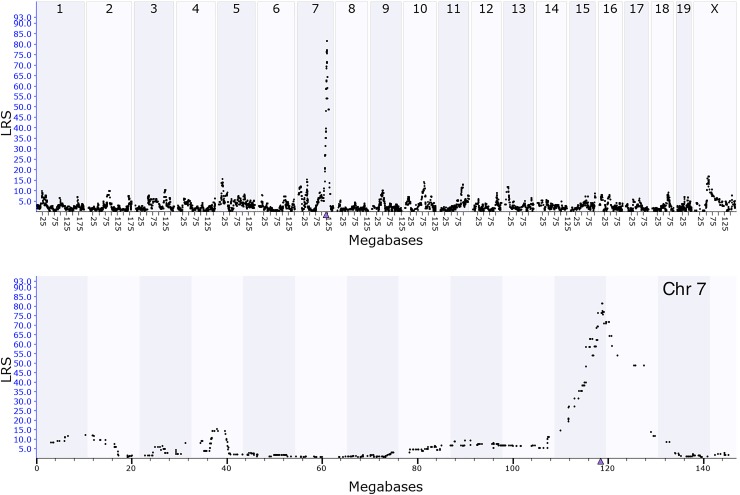
GEMMA eQTL mapping for *Coq7* expression in hippocampus. The *x*-axis represents positions on the mouse genome in megabases (Mb), while the *y*-axis represents the likelihood ratio statistic scores (LRS), a measurement of the linkage between expression of *Coq7* and inheritance of B6 and D2 alleles at different regions of the genome. Top panel shows interval mapping for the whole mouse genome, indicating a significant eQTL at chromosome 7. Bottom panel shows a close-up view of chromosome 7 in which the peak of eQTL was around 119 Mb of chromosome 7 where *Coq7* is located (triangle).

The Affymetrix M430 database was used for eQTL mapping. *Coq7* is located on chromosome 7 at 118.53 Mb, and a significant eQTL modulating the expression of this gene with a likelihood ratio statistics (LRS) of 81 was mapped to the *Coq7* gene locus (Figure 2). This indicates that *Coq7* is *cis-*regulated, meaning one or more sequence variants affecting its expression is located within or near the gene itself. Using the open access sequence data resources at Sanger^5^, we identified 140 SNPs in *Coq7* between the BXD parental strains B6 and D2. Eleven SNPs are located at 3′-UTR and coding area (Table 1) including one stop gain variance that could have strong downstream effect. The rest of them are located at non-coding area. All SNPs are listed in Supplementary Table 1. In addition, we identified 42 indels in *Coq7* between the BXD parental strains (Supplementary Table 2), with two of them being frameshift variants that could also have a strong downstream effect.

### Expression Differences of *Coq7* Between Saline and Ethanol Groups

We used the Affymetrix MTA datasets to analyze the effect of alcohol on the expression of *Coq7* in the hippocampal tissue of B6 and D2 mice using an ANOVA with treatment, strain and sex as the between subject factors. The average of *Coq7* expression in the saline group was 7.46 ± 0.01 (log2 scale), while the average for the ethanol group was 7.52 ± 0.01 (log2 scale), indicating an increase in expression after ethanol treatment. ANCOVA analysis showed a significant effect of ethanol treatment on *Coq7* transcript abundance (*P* = 0.029) and also interaction of ethanol treatment and sex significantly effects on *Coq7* transcript abundance (*P* = 0.002) (Table 2). For B6 strain, the average of Coq7 expression in the ethanol group was 7.53 ± 0.04 (log2 scale), while the average for the saline group was 7.46 ± 0.03 (log2 scale). For D2 strain, the average of Coq7 expression in the ethanol group was 7.51 ± 0.05 (log2 scale), while the average for the saline group was 7.47 ± 0.03 (log2 scale). The *T*-test analysis showed a significant effect of ethanol treatment on *Coq7* transcript abundance in the B6 strain (*P* = 0.0234), not in the D2 strain (*P* = 0.2454).

**Table 2:** ANOVA analysis of *Coq7* expression in the hippocampus.

Source	df	Sums of Squares	Mean Square	*F*-ratio	*P*-value
Intercept	1	1123.54	1123.54	3839480	<0.0001
Treatment	1	0.003281	0.003281	11.213	0.0286
Strain	1	0.000225	0.000225	0.7684	0.4302
Treatment^∗^Strain	1	0.001315	0.001315	4.4951	0.1013
Sex	1	0.000281	0.000281	0.9592	0.3828
Treatment^∗^sex	1	0.013775	0.013775	47.073	0.0024
Strain^∗^Sex	1	0.000037	0.000037	0.12496	0.7416
Age	1	0.000455	0.000455	1.5543	0.2805
Treatment^∗^Age	1	0.001535	0.001535	5.2472	0.0838
Strain^∗^Age	1	0.001828	0.001828	6.2477	0.0668
Sex^∗^Age	1	0.001784	0.001784	6.097	0.069
Body Weight (Wt)	1	0.002022	0.002022	6.9113	0.0583
Treatment^∗^BodyWt	1	0.000328	0.000328	1.1205	0.3495
Strain^∗^BodyWt	1	0.000149	0.000149	0.50948	0.5148
Sex^∗^BodyWt	1	0.000631	0.000631	2.1555	0.216
Age^∗^BodyWt	1	0.001043	0.001043	3.5626	0.1321
Error	4	0.001171	0.000293		
Total	19	0.039494			


### qRT-PCR Validation

*Coq7* expression was validated using RT-PCR. The RT-PCR results showed significantly increased expression of *Coq7* (*F* = 11.77, *P* = 0.027) after ethanol treatment. This was consistent with the results from microarray analysis. In addition, we also found significant strain differences between B6 and D2 (*F* = 9.326, *P* = 0.038), and sex difference between males and females (*F* = 11.788, *P* = 0.027).

### Gene Ontology Analysis

We used the top 500 transcripts co-expressed with *Coq7* from both saline and ethanol group to perform gene ontology (GO) analysis. For the saline-treated group, significant biological processes included “ATP metabolic process,” “mitochondrion organization,” “electron transport chain,” and “ubiquinone biosynthetic process” (Supplementary Table 3). For the ethanol treated group, 54 enriched GO biological processes categories reached significance (Supplementary Table 4). Many of the categories in the ethanol group overlapped with those in the saline group, but all of the ATP metabolic and mitochondrial function related categories were no longer significant. Instead, several new enriched categories including serine metabolic process, behavior and cognition show up.

Gene pathway enrichment analysis for the saline-treated group revealed 21 related pathways (adj *P* < 0.05, Table 3), while the ethanol-treated group revealed six related pathways (adj *P* < 0.05, Table 4). The top enriched pathway in the saline group including “Electron Transport Chain” and “Oxidative phosphorylation” are not shared with the ethanol group. A pathway of interest unique to the ethanol group is “Oxidative stress.”

**Table 3:** Significant pathways for the Saline group.

Pathway name	# of genes	raw *P*-value	adj *P*-value
Electron Transport Chain	24	1.29E-19	6.97E-18
TCA Cycle	13	1.61E-14	4.35E-13
Amino Acid metabolism	19	8.53E-13	1.54E-11
Oxidative phosphorylation	13	9.11E-11	1.23E-09
Glycolysis and Gluconeogenesis	12	1.22E-10	1.32E-09
Kennedy pathway	6	1.58E-07	1.42E-06
Fatty Acid Biosynthesis	6	1.94E-05	0.0001
One carbon metabolism and related pathways	6	0.0004	0.0027
PPAR signaling pathway	8	0.0005	0.003
Nucleotide Metabolism	4	0.0006	0.0032
One Carbon Metabolism	5	0.001	0.0042
Folic Acid Network	4	0.0009	0.0042
Proteasome Degradation	6	0.0011	0.0042
selenium	4	0.001	0.0042
Glutathione and one carbon metabolism	4	0.0045	0.0159
mRNA processing	18	0.0047	0.0159
Acetylcholine Synthesis	2	0.0078	0.0248
Urea cycle and metabolism of amino groups	3	0.0091	0.0273
Triacylglyceride Synthesis	3	0.0103	0.0293
Glutathione metabolism	3	0.0116	0.0313


**Table 4:** Significant pathways for the Ethanol group.

Pathway name	# of genes	raw *P*-value	adj *P*-value
One carbon metabolism and related pathways	7	4.62E-05	0.0027
mRNA processing	20	0.0008	0.0155
Cytoplasmic Ribosomal Proteins	7	0.0008	0.0155
Oxidative Stress	4	0.0021	0.0305
Glutathione and one carbon metabolism	4	0.0043	0.0416
Myometrial Relaxation and Contraction Pathways	9	0.0042	0.0416


### Phenotype Correlation Analysis

**FIGURE 3:**
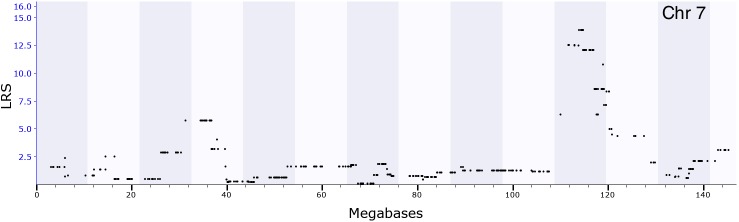
GEMMA QTL mapping for locomotion behavior after ethanol injection (2 g/kg). The *x*-axis represents a position on the mouse genome in megabases (Mb), while the y-axis gives the likelihood ratio statistic scores (LRS). The mapping result showed that the peak of QTL was around 114∼119 Mb of chromosome 7.

We performed correlational analyses with phenotypes archived in the GeneNetwork database to identify ethanol-related behaviors correlated with *Coq7* (*p* < 0.05) expression in the hippocampus. We found 45 ethanol-related phenotypes significantly correlated with *Coq7* expression (Supplementary Table 5), most of which are related to ethanol consumption, ethanol response, ethanol preference, and body temperature after ethanol treatment; some of which are mapped near *Coq7* location. For example, one phenotype (record ID 10496, ethanol response) has a significant QTL with a LRS of 13.8 (*P* < 0.05) on chromosome 7 at 114∼119 Mb (Figure 3) where *Coq7* is located.

## Discussion

Currently, mechanisms through which ethanol affects *Coq7* and ubiquinone are unknown and previous studies analyzing *Coq7* and ethanol are lacking. To this end, we attempted to elucidate the relationship between ethanol and expression of *Coq7* in the hippocampus of BXD mice by identifying genes and biological pathways that may link the two. *Coq7* is variably expressed in naïve hippocampal tissue from BXD RI strains and is *cis-*regulated, making it an excellent candidate for study as a modifier of gene expression or biological phenotypes (Ciobanu et al., 2010). We found that there are multiple SNPs and Indels in *Coq7*, which may be responsible for its differential expression in the hippocampal tissues across BXD strains. This also suggests that one or more polymorphism may affect *Coq7* expression and play a role in gene regulation (Kunugi et al., 2001; Jablonski et al., 2005). Furthermore, we found increased expression of *Coq7* following acute ethanol injection and identified 45 ethanol-related phenotypes correlated with *Coq7*, supporting its likely involvement in ethanol responses (Supplementary Table 5). These findings included six phenotypes related to conditioned taste aversion and ethanol preference. It has been previously proposed that *Coq7* may play a role in alcohol consumption (Tabakoff et al., 2009), aligning with the result of our phenotypic analysis. While this phenotypic alignment is interesting, it should be noted that our experiment did not directly test ethanol consumption behaviors before and after ethanol injection, limiting how generalizable this result may be. Future direct observation of changes in ethanol preferences following ethanol injection or a period of oral consumption would likely be worthwhile.

Comparison of functional enrichment analysis results between our treatment groups may hold insight into why *Coq7* was upregulated following ethanol injection. In the saline treatment group, we found processes related to cellular metabolism and ATP generation (Supplementary Table 3). Presumably, when proteins needed to break down energy substrates are expressed, *Coq7* is also expressed. This is unsurprising considering CoQ’s role in the electron transport chain as an electron shuttle, representing a lynchpin of the final step of ATP generation following metabolism. Conversely, these metabolic functions were largely insignificant in the ethanol treatment group’s analysis, suggesting a less prominent role. This may well be representative of ethanol’s effect on the mitochondria, which has been shown to decrease respiratory rates and the rate of ATP synthesis following chronic exposure (Thayer and Rubin, 1979; Manzo-Avalos and Saavedra-Molina, 2010). This notion was further corroborated by pathway enrichment analysis, which found pathways such “Electron Transport Chain,” “TCA cycle,” “Amino Acid Cycle,” “Oxidative phosphorylation,” and “Glycolysis and Gluconeogenesis” associated with the saline treated group (Table 3), but not the ethanol treatment group.

The ethanol group uniquely had “Oxidative Stress” as a significantly enriched gene pathway (Table 4). Notably, three of the four genes in this gene pathway encode proteins contributing to anti-oxidant enzymes: *Sod2* (superoxide dismutase, Azadmanesh and Borgstahl, 2018), *Gclc* (glutamate-cysteine ligase, a catalyst for glutathione synthesis; Lu, 2009, 2013), and *Txnrd1* (thioredoxin reductase 1; Turanov et al., 2010). While all three are well-established anti-oxidants, previous work on their interactions with ethanol vary greatly. Superoxide dismutase (SOD) is perhaps best studied but also the most controversial. SOD activity in the murine brain following ethanol exposure has been shown to increase (Somani et al., 1996; Enache et al., 2008; Reis et al., 2017), remain unchanged (Gönenç et al., 2005) and decrease (Ledig et al., 1981) under various conditions. The other two genes are less well characterized. *Gclc* mRNA expression shown to be induced by ethanol in rat liver and brain (Lu et al., 1999; Narasimhan et al., 2011) and *Txnrd1* has not been as well studied in terms of ethanol-induced stress.

However, a recent paper by Casañas-Sánchez et al. (2016) found all three of these genes, as well as many other anti-oxidant genes, to have increased expression in hippocampal derived HT22 cells following acute, sub-toxic ethanol exposure. To explain its result, this paper emphasized the growing understanding of ROS as second messengers, capable of influencing gene expression (D’Autréaux and Toledano, 2007; Kaspar et al., 2009; Schieber and Chandel, 2014). These findings have been pioneered in the growing field of “redox biology,” which heavily emphasizes a more nuanced understanding of the impact of ROS. Current work suggests that varying levels of ROS production (so called “basal,” “low,” “intermediate,” and “high” oxidative stress) cause different cellular reactions (Lushchak, 2014; Sies, 2015). Casañas-Sánchez et al. (2016) reasoned that low levels of ethanol increased the level of oxidative stress, as ethanol is a well-documented potent producer of ROS by virtue of its metabolism (Lieber, 1976; Zakhari, 2006; Das and Vasudevan, 2007), resulting in beneficial mitohormetic anti-oxidant gene expression.

This phenomenon may similarly be occurring in our study, as evidenced by the induction of *Coq7* and the phenotypic shift to oxidative stress protection in the ethanol treatment group. It may be that CoQ is unimportant as an anti-oxidant under basal or low-level oxidative stress, as seen in CoQ knockout mouse studies, but is induced under heightened oxidative stress levels alongside other well-known anti-oxidants. The variation of *Coq7* expression in BXD RI mice could result in variation in this inducible response, accounting for differences in how individual strains of BXDs react to ethanol ingestion. Tabakoff et al. (2009) argue that several of their identified “alcohol consumption genes” could theoretically modulate GABA release from the hypothalamus to areas like the ventral tegmental area, ultimately affecting downstream reward behaviors through a variety of molecular mechanisms (see their Figure 3). Logically, the variation in genetic expression among individuals or under different conditions may end up modulating behavior. While Tabakoff mentions *Coq7* as an identified “ethanol consumption gene,” they do not outline how it might affect GABA release in this way. With the idea of ROS as signaling molecules in mind, variation in *Coq7* expression (or other anti-oxidant defenses) could limit or permit ROS signaling, potentially altering critical neurologic signaling pathways related to reward-seeking behavior, similar to those proposed by Tabakoff et al. (2009). How these alterations would occur is still a matter of investigation. Work has examined some of the intracellular mechanisms by which ROS can affect plasticity and signaling in the hippocampus, mainly through induction of long-term potentiation (LTP). This induction seems to be through ROS acting as a second messenger, inducing phosphorylation of established LTP effectors such as PKC, ERK, and CamKII (Malinow et al., 1989; Klann et al., 1998; Kishida et al., 2005). For recent reviews on the topic, please see Beckhauser et al. (2016) and Oswald et al. (2018). However, there has been little investigation into ROS’s effect on motivational circuitry. While further work is needed, ethanol-induced oxidative stress may represent a worthwhile line of inquiry to better understand hippopcampal cellular responses to heightened oxidative stress *in vivo* and their downstream effects on neurotransmission and behavior.

In sum, we analyzed the effect of ethanol on the expression of *Coq7* using a systems genetic approach. We identified an eQTL showing variability in *Coq7* expression in BXD RI mice and found its expression to be increased following ethanol treatment. We also uncovered several pathways and genes which may interact with *Coq7* to regulate the ethanol response. Based on this and previous reports, *Coq7* may act as an inducible anti-oxidant at heightened levels of oxidative stress, aligning both with previous work and current questions on this ubiquitous molecule. Further research into the specific interactions between *Coq7* and identified genes may elucidate their relationships and shed light on how *Coq7* affects mitochondria following acute ethanol consumption.

## Ethics Statement

All animal work was conducted in accordance with of and procedures approved by the Institutional Animal Care and Use Committees at The University of Tennessee Health Science Center and University of Memphis following NIH guidelines.

## Author Contributions

DZ provided the primary analysis and primary writing for the manuscript. YZ provided the primary interpretation of experiment and primary writing for the manuscript. MH provided the primary writing, editing, literature review, and for the manuscript and data interpretation of experiment. WZ provided the analysis and RT-PCR experiment. AS-D, MC, AD, and BJ provided the final editing and critique of manuscript. KH provided the primary experiment, final editing and critique of the manuscript. LL provided the procedure, planning, funding, and oversight for experiment as well as final editing and critique of manuscript.

## Conflict of Interest Statement

The authors declare that the research was conducted in the absence of any commercial or financial relationships that could be construed as a potential conflict of interest.

## Footnotes

1 www.tel-test.com

2 http://www.nanodrop.com

3 https://www.roche-applied-science.com

4 www.genenetwork.org

5 https://www.sanger.ac.uk/sanger/Mouse_SnpViewer/rel-1303

6 http://bioinfo.vanderbilt.edu/webgestalt/
